# Pseudomyopathic Changes in Needle Electromyography in Lambert-Eaton Myasthenic Syndrome

**DOI:** 10.1155/2013/369278

**Published:** 2013-07-18

**Authors:** Teppei Komatsu, Kota Bokuda, Toshio Shimizu, Tetsuo Komori, Reiji Koide

**Affiliations:** ^1^Department of Neurology, Tokyo Metropolitan Neurological Hospital, 2-6-1 Musashidai, Fuchu, Tokyo 183-0042, Japan; ^2^Department of Neurology, The Jikei University School of Medicine, Japan; ^3^Department of Neurology, National Hakone Hospital, Japan

## Abstract

Lambert-Eaton myasthenic syndrome (LEMS) is a rare presynaptic disorder of the neuromuscular junction in association with cancer and subsequently in cases in which no neoplasm has been detected (O'Neill et al., 1988). The diagnosis of LEMS is based on the combination of fluctuating muscle weakness, diminished or absent reflexes, and a more than 60% increment of compound muscle action potential (CMAP) amplitude after brief exercise or 50 Hz stimulation for 1 s in a repetitive nerve stimulation (RNS) test (Oh et al., 2005). On the other hand, needle electromyography (EMG) findings related to LEMS have not been well described. Here, we report a case of LEMS, which showed apparent myopathic changes in needle EMG findings. Furthermore, we retrospectively examined the needle EMG findings in 8 patients with LEMS. In six of the 8 patients, the EMG findings showed myopathy-like findings. Although the findings of needle EMG indicated myopathic changes at a glance, the motor unit potential (MUP) returned to normal after a sustained strong muscle contraction. We propose the name “pseudomyopathic changes” for this phenomenon.

## 1. Case Report

**Figure 1:**
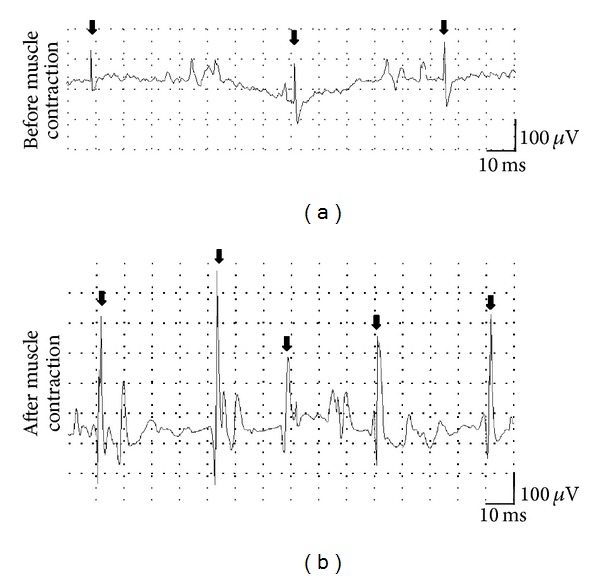
Needle EMG findings in patient 1. The MUPs (arrows) with short durations and low amplitudes were observed in the biceps brachii muscle (a). After a strong muscle contraction for 10 s, the MUP sizes were normalized (b).

A 69-year-old female (patient 1 in Table 1) developed a gait disturbance 6 months before admission and suffered from the gradual progression of weakness in her extremities. On admission, neurological examination showed proximal muscle weakness and reduced deep tendon reflex. Routine laboratory test results were unremarkable. Antinuclear antibodies, anti-acetylcholine (ACh) receptor antibody, and anti-voltage-gated calcium channel (VGCC) antibodies were negative. The results of chest and abdominal CT were unremarkable. In a nerve conduction study, CMAPs had slightly low amplitudes, and nerve conduction velocities were normal in the median and ulnar nerves. Needle EMG showed early recruitment and polyphasic MUPs with short durations and low amplitudes, suggesting the diagnosis of myopathy. Interestingly, the myopathic EMG findings were improved after sustained strong muscle contraction for 10 s; the MUP sizes returned to normal after a sustained strong muscle contraction, and the early recruitment of MUPs in the weak contraction was clearly normalized in the biceps brachii muscle (Figure 1). Thereafter, we performed an RNS test because she complained of easy fatigability. The RNS test showed an obvious incremental response of the CMAP amplitude of the abductor digiti minimi muscle after high-rate stimulation of the ulnar nerve, indicating the diagnosis of LEMS [1–3]. We examined the patient for systemic malignancies, and the endoscopic examination of the colon revealed adenocarcinoma in the sigmoid colon. Her muscle weakness slightly improved after resection of the colon cancer.

## 2. Discussion

We retrospectively analyzed the needle EMG findings in the 8 patients with LEMS (Table 1). All patients were hospitalized in our hospital between June 1997 and August 2012. The mean age was 66 years (range from 49 to 72). Malignant tumors were detected in six of the 8 patients, of whom five had small-cell lung cancer (SCLC) and one had colon adenocarcinoma. Anti-VGCC antibodies were detected in all five patients with LEMS associated with SCLC. Incremental responses in the RNS test were observed in all patients. In six of the 8 patients, myopathy-like findings similar to the present case were found by needle EMG, characterized by initial myopathic patterns and normalization of the recruitments and MUP amplitude after a strong muscle contraction. We propose the name “pseudomyopathic changes” for this phenomenon. 

LEMS is typically caused by pathogenic autoantibodies to the presynaptic VGCCs in the membrane of motor nerve terminals, inducing the impairment of ACh release [2, 4]. Mechanism of abnormalities in the needle EMG of LEMS patients is presumed as follows: end-plate potential (EPP) decreases in LEMS because EPP increases in proportion to the number of Ach quanta liberated from the nerve terminal. When the EPP fails to reach excitability threshold of the muscle cell, an action potential does not ensue and a neuromuscular block results. Desynchronized discharges and the intermittent blocking of individual muscle fibers within the motor unit induce polyphasic MUP with a low amplitude, mimicking myopathic features of EMG. Normalization of the MUP amplitude and the motor unit recruitment after a strong muscle contraction is attributed to the enhancement of ACh release from the nerve terminals. 

Physicians should not easily eliminate the possibility of LEMS, if myopathic features of EMG were observed in the patients with muscle weakness. 

## Figures and Tables

**Table 1:** Characteristics of patients.

Patient	Sex	Age	Needle EMG	RNS	Neoplasm	Anti-VGCC antibody
1	F	69	Pseudomyopathic	Increased	Colon adenocarcinoma	Negative
2	M	70	Pseudomyopathic	Increased	SCLC	Positive
3	F	72	Pseudomyopathic	Increased	SCLC	Positive
4	M	71	Pseudomyopathic	Increased	SCLC	Positive
5	M	49	Pseudomyopathic	Increased	Unknown	Positive
6	M	67	Pseudomyopathic	Increased	SCLC	Positive
7	F	56	Normal	Increased	SCLC	Positive
8	M	71	Normal	Increased	Unknown	Unknown

EMG: electromyography; RNS: repetitive nerve stimulation; SCLC: small-cell lung carcinoma; VGCC: voltage-gated calcium channel.
